# Long-Term Exposure to Ionizing Radiation from the Semipalatinsk Nuclear Test Site and Risk of Cardiovascular Mortality

**DOI:** 10.3390/ijerph22121781

**Published:** 2025-11-25

**Authors:** Altay Dyussupov, Dariya Shabdarbayeva, Nailya Chaizhunussova, Andrey Orekhov, Galiya Alibayeva, Assel Baibussinova, Madina Abenova, Meruyert Massabayeva, Alexandra Lipikhina, Gulshara Abdildinova, Zhanargul Smailova, Saulesh Apbassova, Dinara Mukanova, Saule Kozhanova, Ernar Kairkhanov, Diana Ygiyeva, Raushan Dosmagambetova, Asset Izdenov, Lyudmila Pivina

**Affiliations:** 1 University Board, Semey Medical University, Semey 071400, Kazakhstandosmagambetova@qmu.kz (R.D.);; 2 Department of Public Health, Semey Medical University, Semey 071400, Kazakhstan; 3 Therapy Department, Semey Medical University, Semey 071400, Kazakhstan; 4 Department of Epidemiology and Biostatistics, Semey Medical University, Semey 071400, Kazakhstan; 5 Center of Scientific-Research Laboratory, Semey Medical University, Semey 071400, Kazakhstan; meruyert.massabayeva@smu.edu.kz; 6 RI of Radiation Medicine and Ecology, Semey Medical University, Semey 071400, Kazakhstan; a.v.lipikhina@mail.ru; 7 Medical Center Hospital of the President’s Affairs Administration of the Republic of Kazakhstan, Astana 010000, Kazakhstan; 8 Department of Pathological Anatomy and Forensic Medicine, Semey Medical University, Semey 071400, Kazakhstan; 9 Department of Simulation and Educational Technologies, Semey Medical University, Semey 071400, Kazakhstan; dinara.mukanova@smu.edu.kz; 10 Department of Anatomy, Histology and Topographic Anatomy, Semey Medical University, Semey 071400, Kazakhstan; 11 Department of Emergency Medicine, Semey Medical University, Semey 071400, Kazakhstan; 12 Department of Medical Education, Ministry of Healthcare of the Republic of Kazakhstan, Astana 010000, Kazakhstan; asetizdenov@mail.ru

**Keywords:** Semipalatinsk nuclear test site, cardiovascular mortality, epidemiology, radiation hazards, radioactivity

## Abstract

**Background**: Environmental problems can significantly influence population health. Cardiovascular diseases (CVDs) are the most common cause of death in developed countries including in Kazakhstan. The aim of our study was to assess the relationship between exposure to ionizing radiation and mortality risks from major CVDs for the population of Kazakhstan living in areas adjacent to the Semipalatinsk nuclear test site (SNTS). **Materials and Methods**: The study of the structure and dynamics of cardiovascular mortality was based on the State Scientific Automated Medical Registry (SSAMR) database. Among members of the exposed group, the median equivalent radiation dose was 864.0 mSv, compared to 64.4 mSv in the control group. It should be noted that almost the entire population of the Semipalatinsk region was exposed to some degree of radiation; however, the Kokpekti district received the lowest radiation doses. **Results**: Mortality rates from CVD were statistically significantly higher in the radiation-exposed population from 1960 to 1994, with RR fluctuating from 1.118 to 8.7. The predominant mortality events were chronic coronary heart disease, chronic cerebrovascular disease, and hemorrhagic stroke throughout the study period, and acute myocardial infarction within 20 years of the start of nuclear testing. In the exposed group, RR = 10.35 for chronic cerebrovascular disease, RR = 3.56 for hemorrhagic stroke, and RR = 5.77 for peripheral arterial atherosclerosis. A dose of 100–500 mSv increased the risk of mortality from CVD by 3.14 times, and a dose of >600 mSv increased it by 7.05 times. **Conclusions**: A link has been established between long-term exposure to ionizing radiation and increased risks of mortality from CVD in the population of areas contaminated with radiation as a result of nuclear testing.

## 1. Introduction

The decision to establish the Semipalatinsk Nuclear Test Site (SNTS) was made in the USSR on 21 August 1947. The site was located in northeastern Kazakhstan, primarily in the Semipalatinsk region, but also encompassing parts of the Pavlodar and Karaganda regions. Over the course of 40 years, 458 nuclear explosions were detonated at the Semipalatinsk Nuclear Test Site, including 30 above-ground, 118 atmospheric, and 470 underground explosions [1]. The main type of exposure of the population was repeated external gamma irradiation due to the passage of a radioactive cloud and radioactive fallout, as well as subsequent chronic internal irradiation due to radionuclides entering the human body with food, drinking water and inhaled air [2].

There are no comparable radiation situations in the world to those that developed in the territories of Kazakhstan adjacent to the Semipalatinsk nuclear test site. Cumulative absorbed and effective equivalent doses of radiation to the population during the testing period (1949–1965) resulted from acute external and long-term chronic internal irradiation in varying dose ranges [3]. Given the long period of time since the tests began, we do not have comprehensive data characterizing the acute and immediate effects of radiation exposure on the population living in the areas surrounding the test site [4]. Therefore, an assessment of the medical and social consequences of nuclear weapons testing is possible through an analysis of retrospective information. The most accurate data in this regard may be those characterizing mortality rates over time in the groups exposed to radiation [5].

Due to the secrecy of data on the radiation doses of residents of radiation-exposed areas, a comprehensive analysis of the medical consequences of population exposure to radiation became possible only in the early 1990s, when some information was obtained on the chronology of nuclear explosions, the radiation-hygienic situation in the studied areas, and effective and collective doses for part of the population were reconstructed [6].

To study the health consequences of radiation exposure in Kazakhstan, the most objective and accessible information was data on causes of death for the entire period of nuclear testing and in subsequent years up to the present. Information on deaths in the Republic of Kazakhstan is available in two forms: medical death certificates issued by medical institutions to relatives of the deceased and death certificates registered as part of civil registration. Furthermore, regional statistical offices conduct annual mortality analyses based on these documents [7].

Beginning in 1957, Dispensary No. 4, now the Research Institute of Radiation Medicine and Ecology, began collecting information on the population living in radiation-exposed settlements near the Semipalatinsk test site, their health status, and the causes of death. This information formed the basis for the creation of the State Scientific Automated Medical Registry of Residents of Kazakhstan Exposed to Radiation (SSAMR) [7]. The process of collecting and updating information on individuals exposed to radiation continues to this day, reflected in constant changes to the registry database. Assessing the medical consequences of ionizing radiation requires calculating the radiation doses to the population, which allows for the interpretation of the nature and extent of radiation effects.

Cause-of-death information provides the basis for large-scale epidemiological studies, enabling the assessment of demographic indicators in a country or region, the causes and risk factors for deteriorating health in large populations, including environmental factors, and the development of state programs to prevent environmentally related diseases. Studying and analyzing population mortality trends allows us to assess the complex interactions between such structures as the state of the healthcare system as a whole, lifestyle changes over time, environmental conditions, and the social conditions of citizens [8].

It is known that in all countries of the world, over the past decades, diseases of the circulatory system have predominated in the mortality structure, which is associated, first of all, with an increase in life expectancy, and secondly, with an increasing contribution of such traditional risk factors for CVD as arterial hypertension, diabetes mellitus, obesity and dyslipidemia, as well as behavioral disorders, in particular, smoking [9]. The factor of radiation exposure in relation to the development of CVD and mortality has been studied much less. The first data on the increased risks of cardiovascular diseases with exposure to ionizing radiation were obtained in cohorts of people who survived the atomic bombing in Japan [10,11,12]. Using the example of the Life Span Study cohort, a significant positive association was established between the radiation dose and mortality from cardiovascular diseases for the period from 1950 to 2008 [13]. Significantly high risks of excess mortality from CVD were found in cohorts of individuals exposed to radiation in occupational settings [14,15].

Very few studies on circulatory diseases have been conducted in Kazakhstani residents affected by the activities of the Semipalatinsk Nuclear Test Site, and these have mainly focused on the incidence and prevalence of individual subtypes of diseases, such as arterial hypertension and cerebrovascular accidents [16,17,18]. Only one article, written within the framework of an international collaboration, was devoted to mortality from circulatory diseases in the historical cohort of residents of the Semipalatinsk region exposed to radiation for the period from 1960 to 1999 [19].

The aim of this study was to assess the relationship between exposure to ionizing radiation and the risk of mortality from major cardiovascular diseases for the population of Kazakhstan living in areas adjacent to the Semipalatinsk nuclear test site.

## 2. Materials and Methods

### 2.1. General Information About SSAMR

A study of the dynamics and structure of mortality rates among residents of the Semipalatinsk region adjacent to the test site is based on the SSAMR database. This registry is designed to maintain a long-term personal record of individuals directly exposed to radiation and their descendants across multiple generations, calculate individual equivalent radiation doses, monitor and assess the health status of individuals included in the registry, study the impact of radiation and non-radiation factors on morbidity and mortality in the exposed population, and formulate strategies to minimize the impact of ionizing radiation on the health of the exposed population. the registry was created in 2002 in collaboration with Japanese colleagues from the Radiation Effect Research Foundation and with the support of the Ministry of Health of the Republic of Kazakhstan [20,21].

Currently, the SSAMR database contains information on over 374,000 individuals, including individuals with varying vital statuses. This information includes passport data, periods of residence in radiation-contaminated areas (which allows for the calculation of equivalent radiation doses), vital status, education, occupation, medical information based on the results of a comprehensive or screening examination, and, in the event of death, the death certificate number and cause of death. Each individual included in the registry database is assigned a unique number, which allows access to all information available on that individual [7].

### 2.2. Calculation and Assessment of Radiation Doses for Individuals Included in the SSARM

The SSARM has implemented an automated program for calculating individual radiation doses in accordance with the methodological recommendations adopted in Kazakhstan [22]. The assessment is conducted in accordance with the classification of territories by degree of radiation risk affected by the test site [23]. The presented methodology for calculating the equivalent radiation dose includes an assessment of the contribution of such components as the power of atmospheric and underground explosions conducted over the entire period of the test site’s operation; the altitude of the explosion, wind speed, climatic characteristics at the time of the test, the location of the trace of the radioactive cloud, as well as the duration of residence in each specific radiation-contaminated area. This methodology is based on the joint US-Russian dose reconstruction methodology, developed based on the shared experience of dose reconstruction experts [24,25,26,27]. An individual equivalent radiation dose was calculated for each member of the SSARM. The median equivalent radiation dose for members of the exposed group (residents of the Abay and Beskaragay districts of the former Semipalatinsk region) was 864 mSv (IQR 604 mSv), while in the control group this value was 64.4 mSv (IQR 46.3 mSv).

The study was approved by the Ethics Committee of Semey Medical University (protocol №2b, 18 December 2024)

### 2.3. Characteristics of Study Groups from the SSARM Mortality Subregistry

Inclusion criteria for the study group: residents of the Abaysky District (the villages of Karaul, Sarzhal, and Kainar) and the Beskaragaysky District (the villages of Dolon, Mostik, and Cheremushka) who died at various times and who lived in the study villages at any time during the nuclear testing period (1949–1989) and were exposed to maximum radiation doses. Deceased descendants of these individuals were also included in the study group. Residents of the Kokpekty District of the Semipalatinsk Region, served as a control group. This area was designated as the control region because its population is comparable to that of the radiation-exposed area in all cardiovascular risk factors except for radiation exposure. It should be noted that almost the entire population of the Semipalatinsk region was exposed to some degree of radiation; however, the Kokpekti district received the lowest radiation doses. The subregister contains information from 1949 to 2024.

All members of the subregistry have the vital status of “deceased,” and the date and/or cause of death are recorded. Information on the date and cause of death was verified in the regional archives of civil registration offices (ZAGS), the Integrated Medical Information System of the Republic of Kazakhstan, and the governing bodies of the localities where the deceased citizens resided.

The mortality subregister currently includes 8580 people from the radiation-exposed settlements and 3743 residents of the Kokpekty district. Of these, 3482 died from circulatory diseases in the exposed group (the study’s main group), and 1886 in the control group.

For each subregistry member, information was included on the date of birth and death, age at death, gender, nationality, occupation, death code according to the International Classification of Diseases, 10th Revision (ICD-10), place of residence, and radiation dose. Cause of death coding was performed by medical professionals.

To maintain confidentiality, each member of the subregistry is assigned a unique identification number, Sysid, which corresponds to the registration card number of the individual exposed to radiation in the SSARM database. Population information for the studied settlements was obtained from the National Statistical Bureau of Kazakhstan, as well as from archival data.

### 2.4. Statistical Analysis

Statistical data processing was performed using the SPSS (v.20, IBM Ireland Product Distribution Limited, Dublin, Ireland) and Jamovi (v.2.5, Sydney, Australia) packages. In accordance with traditional approaches, the first stage of the study determined the structure of the mortality rate for all causes for the period from 1949 to 2024. Subsequently, in accordance with the stated goal, intensive mortality rates per 100,000 population for causes of CVD were calculated for the exposed and unexposed study groups. Mortality was also analyzed by time periods (1949–1954, 1955–1959, etc., up to 2020–2024). Linear regression methods and Joinpoint analysis (NCI Joinpoint Regression Program, v.5.0) were used to identify trends.

When comparing mortality rates in the study groups, the Pearson χ^2^ test was used to compare proportions (for individual nosological forms of CVD); the nonparametric Mann–Whitney test (in case of violation of normal distribution) was used to compare quantitative indicators.

Age-standardized mortality rates (ASMRs) were calculated using the direct standardization method, with the European standard population as the reference.

In the second stage of the analysis, relative risks (RR) and 95% confidence intervals (95% CI) were calculated to compare the probability of death from CVD between districts. To assess the relationship between mortality, the dose of ionizing radiation, and district affiliation, Cox regression and logistic regression were used to estimate the hazard ratios (HR) with their 95% CI. Mortality dynamics were forecast using the R statistical package (version 4.3.2). The critical level of statistical significance was set at *p* < 0.05. Approximation quality indicators (R^2^, AIC, BIC) were additionally specified for the forecast models.

## 3. Results

An analysis of the overall mortality structure in the study groups revealed a significant excess of deaths from infectious diseases, cancer, nervous system pathologies, respiratory diseases, and congenital malformations in the radiation-exposed group, while deaths from circulatory diseases, injuries, and suicides were more common in the unexposed group. Overall, the rates in the exposed and control groups differed statistically significantly (Table 1).

Table 2 presents the dynamics of mortality rates from CVD during the study period and its age structure. In both study groups, men predominated (53.4% in the exposed group, 52.4% in the control group), but no statistically significant differences were noted. No significant differences in the age structure of mortality in the study groups were found. In the dynamics of mortality rates from cardiovascular disease, attention is drawn to a statistically significant increase in intensive rates in the exposed group, starting in 1960, 10 years after the start of nuclear weapons testing, for 20 years, up to 1980. In subsequent time periods, no significant differences were found in the study groups. From 1949 to 1959, there was an excess of mortality rates in the exposed group from cardiovascular disease in the group with a median age of 3 and 5 years, respectively, for five-year periods. This was probably due to the predominance of children with congenital malformations of the cardiovascular system in the exposed group. Starting from 1960 and in subsequent time periods, the median age in the exposed group ranged from 60 to 73 years old (2020–2024). In the control group, the median age in the first two time periods was 16 years. In subsequent years, no significant differences in median age were found between the study groups.

According to Table 3, the intensive mortality rates per 100,000 population in the radiation-exposed group were statistically significantly higher than in the control group during the period from 1960–1965 to 1990–1994, with the relative risk of mortality fluctuating from 1.18 in 1980–1984 to 8.7 during the period 1960–1964.

**Figure 1:**
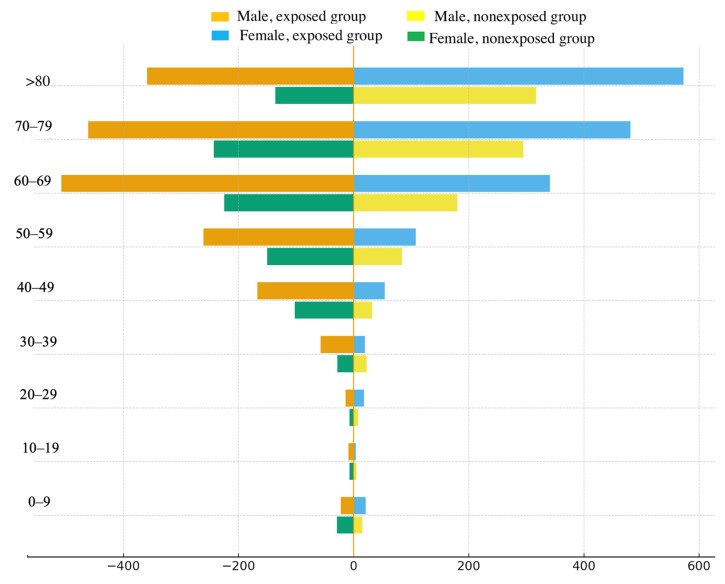
Age and sex pyramid of mortality rates in the study groups.

Figure 1 shows a pyramid of mortality rates for the study groups by age and gender. The data are consistent with the results presented in Table 3. In the exposed group, both men and women exhibited higher mortality rates than unexposed individuals in age groups over 30 years. In both study groups, the number of deaths among men in age groups up to 69 years was higher than among women; in the 70–79 age group, these rates were equal; and in groups over 80 years, mortality rates were higher among women.

When analyzing the mortality structure for individual categories and individual CVDs over time, we selected three time periods corresponding to nuclear weapons testing. The time period from 1949 to 1969 corresponded to the period of atmospheric and above-ground nuclear weapons testing, taking into account the fact that mortality rates may remain elevated for several years after the end of this period. The second time period corresponded to the period of underground nuclear testing, with the addition of a five-year period during which mortality rates could remain elevated. The third time period began from 1995 to the present and corresponded to the period following the closure of the test site. It was found that during the second and third periods of the study, statistically significant differences were observed in the study groups with respect to chronic coronary heart disease and chronic cerebrovascular diseases, with a predominance of indicators in the exposed group; with respect to peripheral atherosclerosis, this trend was noted only in the period from 1970 to 1994; for acute myocardial infarction, a significant excess of the indicator in the exposed group was established in the first period of the study, from 1949 to 1969, and for hypertension after 1995. With respect to hemorrhagic strokes, a statistically significant predominance of indicators was found in the exposed group for the entire study period; for ischemic strokes, such a trend was found after 1970 until the end of the study period, and for congenital heart defects, they were characteristic only of the period after the cessation of nuclear weapons testing (Table 4).

**Figure 2:**
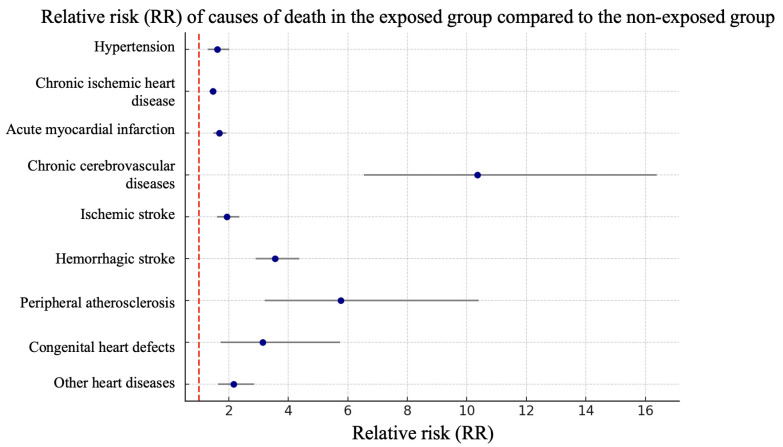
Forest plot of relative risks by cause of mortality in the exposed and unexposed study groups.

Analysis of relative risks of mortality for individual diseases or groups of diseases demonstrated a statistically significant increase in the exposed group for chronic cerebrovascular diseases—10.35 (95% CI 6.54–16.38), hemorrhagic strokes—3.56 (95% CI 2.9–4.36), peripheral arterial atherosclerosis—5.77 (95% CI 3.2–10.4) and congenital heart disease—3.14 (1.72–5.73). The relative risk for hypertension was 1.61 (95% CI 1.29–2.02), for coronary heart disease—1.46 (95% CI 1.35–1.58) (according to Figure 2).

Our attempt to correlate the effects with radiation doses using Cox regression showed that an equivalent radiation dose of 100–500 mSv increased the overall risk of CVD mortality by 3.14 times, while a dose exceeding 600 mSv increased the risk by 7.05 times. The predictive accuracy of this model was 62.4% (Harrell C-index = 0.624) (Table 5).

## 4. Discussion

An analysis of the results of our study allowed us to conclude that in both the radiation-exposed and control population groups, the proportion of mortality from CVD occupied the first place in the structure of causes of death, while in the exposed group, a continuous increase was observed for the proportion of mortality from CVD over 20 years: from 1960 (11 years after the start of nuclear weapons testing) to 1980. Intensive mortality rates from CVD also statistically significantly prevailed among the population exposed to radiation during the period from 1960 to 1994, with RR fluctuations from 1.118 to 8.7.

When assessing the mortality structure for individual CVD categories in the exposed group, a significant increase in the proportion of coronary heart disease, chronic cerebrovascular diseases and hemorrhagic strokes was found throughout the study, ischemic strokes in the period after the cessation of nuclear testing, and acute myocardial infarction within 20 years from the start of nuclear weapons testing.

In the exposed group, the highest relative risks were for chronic cerebrovascular disease (10.35), hemorrhagic stroke (3.56), and peripheral vascular atherosclerosis (5.77). Statistical significance for the relative risks was maintained for virtually all causes of CVD death studied.

Multivariate regression analysis showed a direct relationship between radiation doses and the odds ratio for CVD mortality, but the analysis was conducted without excluding factors other than radiation. Analysis of time series and forecasting of CVD mortality trends in the study groups demonstrated stability of rates up to 2050.

To assess the mortality rates from cardiovascular diseases in the study groups, we attempted to compare them with the mortality rates from cardiovascular diseases in the Republic of Kazakhstan. Thus, in the period from 1960 to 1970, cardiovascular diseases remained in first place in the structure of overall mortality in Kazakhstan, mainly due to ischemic heart disease and cerebrovascular diseases, in particular strokes. In Eastern Kazakhstan (Semipalatinsk region and adjacent areas), higher mortality rates from diseases of the circulatory system were recorded during this period compared to the national average, which was 240–290 per 100,000 population. In the exposed group studied by us, mortality rates from cardiovascular diseases for this period ranged from 642.71 to 699.23 per 100,000 of the population, while in the control group these rates did not differ from the national average [28].

Comparing our results with data from other similar studies is difficult due to the unique nature of each radiation situation worldwide, which exposed specific groups of people. In most well-studied cases, these groups were professionals—nuclear industry workers, Chernobyl liquidators, uranium miners, individuals exposed for medical purposes, and medical personnel performing radiation-related procedures. Regarding populations exposed to radiation during nuclear weapons testing, most mortality publications focused on residents of the Japanese cities of Hiroshima and Nagasaki, who survived the atomic bombing and were included in the Life Span Study (LSS) cohort. All of these studies are cohort studies that calculated the excess relative rate (ERR) per Gy. Our study had a cross-sectional design, so we did not calculate this parameter. Radiation doses varied significantly for each cohort, from high doses for medical purposes in cancer treatment (up to 50 Gy) [29] to moderate and low doses in some occupational cohorts [30,31], where average radiation doses ranged from 25.2 mSv to 0.45 Gy, or in environmental studies such as the Techa River cohort study, where the average radiation dose was 35 mGy [32]. However, only a small number of studies on the association between radiation exposure and CVD have high-quality individual radiation dose estimates.

A strength of our study was the availability of calculated individual equivalent radiation doses for each member of the mortality subregistry. These doses ranged from 0 to 100 cSv (the range of low and moderate radiation doses), with the average equivalent radiation dose for individuals in the exposed group being 86.4 cSv.

The only attempt to link radiation exposure of large cohorts of people living in areas adjacent to the SNTS with mortality from CVD was a cohort study conducted by Grosche B. et al. in 2011 [19]. Radiation doses in this cohort ranged from 0 to 630 mGy. Overall, mortality rates from CVD in the exposed cohort were high, significantly exceeding those in the unexposed cohort, which is fully consistent with our results. Thus, for members of the exposed cohort of Kazakh nationality, the mortality rate was 840.3 per 100,000 population, and for members of Russian nationality—807.4 per 100,000 population. The relative risks of death in the exposed cohort, adjusted for radiation dose, were 1.56 (95% CI 1.34–1.82) for CVD overall in 1970–1979, 1.57 (95% CI 1.35–1.82) in 1980–1989, and 2.14 (95% CI 1.85–2.47) in 1990–1999. For heart disease, the ratios were 1.46 (95% CI 1.19–1.78); 1.56 (95% CI 1.28–1.9), and 2.28 (95% CI 1.89–2.74), respectively; for stroke, 1.58 (95% CI 1.21–2.07); 1.51 (95% CI 1.15–1.98) and 2.05 (95% CI 1.59–2.65). In our study, the relative risks for heart disease were comparable to the results of the aforementioned study; however, for adverse cerebrovascular events, they were significantly higher, which may be due to the fact that we calculated the relative risks in the current time frame. The authors found a direct dose–response relationship when including both exposed and unexposed cohorts in the analysis, with a more than twofold increase in the risk of death. However, analysis of only the exposed cohort did not reveal such a relationship. The ERR/Gy estimates were insignificant in all cases—both for CVD in general and for heart disease and stroke.

In a study by Azizova T et al. (2022), mortality rates from various circulatory diseases were analyzed among workers in the Russian nuclear industry at the Mayak plant for the period from 1948 to 2018 (the same period as in our study) [30]. No association was found between exposure to gamma radiation at average doses of 0.45 Gy for men and 0.37 Gy for women and individual CVD nosologies, with the exception of ischemic stroke in men [8]. The incidence of cerebrovascular diseases was statistically significantly higher in those workers who received a radiation dose greater than 0.1 Gy compared to those whose dose was lower [33]. In our study, the mean equivalent doses were significantly higher, which may explain the high relative risks for diseases such as hemorrhagic stroke, cerebrovascular disease in general, and peripheral arterial atherosclerosis.

An analysis of CVD mortality in the Japanese LSS cohort from 1950 to 2008 demonstrated a positive dose–response relationship for total CVD (ERR/Gy = 0.14; 95% CI: 0.06–0.22), hypertension (ERR/Gy = 0.36; 95% CI: 0.10–0.68), and chronic heart failure (ERR/Gy = 0.21; 95% CI: 0.07–0.37) [13].

In an international cohort of nuclear industry workers in the UK, USA and France (N = 308,297) with an average external dose of 0.025 Gy, a statistically significant excess relative risk per Sv was found for all CVD (ERR/Sv = 0.22; 90% CI: 0.08–0.37), coronary heart disease (ERR/Sv—0.18 (90% CI: 0.004–0.36)), and acute myocardial infarction (ERR/Sv—0.26 (90% CI: 0.03–0.51)) [31,34]. Some studies have shown a direct positive association between the increased risk of mortality from CVD and the duration of the exposure period, even at an average dose of 8.6 mSv [14].

The results of studies examining the relationship between radiation exposure under various conditions and with varying dose ranges and the risk of cardiovascular mortality indicate a significant diversity of scenarios for the development of radiation situations. Most of these studies include occupational cohorts of nuclear industry workers, uranium enrichment workers, uranium miners, Chernobyl accident liquidators, and others [35]. These cohorts are subject to long-term and careful follow-up, eliminating the possibility of numerous errors in calculating radiation doses and recording adverse cardiovascular events. Significantly fewer such studies have been conducted on individuals who have lived for long periods in areas of radioecological concern. Unlike Kazakhstani citizens living around the Semipalatinsk nuclear test site, Japanese residents who survived the nuclear bombings were exposed to a single acute gamma radiation dose; they were relocated from areas contaminated with radionuclides, which helped mitigate their radiation doses. Subsequently, these individuals were subject to careful health monitoring, follow-up, and rehabilitation [35]. In Kazakhstan, residents exposed to radiation continued to reside in contaminated areas until the test site’s closure. Monitoring of their status and health was sporadic and not comprehensive. The creation of the SSAMR, which included all available information on residents of Kazakhstan affected by irradiation, was only initiated in the early 2000s, 10 years after the SNTS closure. Therefore, the information was retrospective and was extracted from death certificates stored in the archives of regional statistical offices, which were received there from medical institutions.

Another limitation of our study is the very long period of study, from 1949 to the present, which is undoubtedly related to problems with diagnostic approaches to various diseases, diagnostic capabilities in establishing the cause of death at different observation periods, and low access to medical care in the early stages of the study. These problems may be related to some errors in disease coding, since death certificates were often issued by paramedics until the 1960s. In 1953, Semipalatinsk Medical University was opened, and its first graduates began working in the late 1950s, which improved the quality of both diagnostics and the accuracy of disease coding. Furthermore, over such a long study period, the cause of death coding system has changed several times. For example, until 1980, Kazakhstan used the ICD-8 system, then replaced it with the ICD-9 system, which was used in Kazakhstan until 1 January 2010, when ICD-10 was introduced.

Since the study groups were comparable in terms of age, sex, dietary habits, and other traditional risk factors for cardiovascular disease, we focused our attention on assessing the association between radiation exposure and cardiovascular mortality.

Despite limitations, our study represents the first attempt to assess the relationship between long-term external and internal radiation exposure in the medium and low dose range and mortality rates from CVD among Kazakhstani citizens permanently residing in areas adjacent to the STNS, compared to unexposed people. The results allowed us to determine the relative importance of separate diseases within the overall structure of CVD as causes of death in exposed people, as well as mortality rates by study period, calculate relative risks for individual circulatory diseases categories and for individual significant diseases, determine the relationship between the risk of CVD mortality and radiation doses, and forecast for CVD mortality rates through 2050.

## 5. Conclusions

The results of our study suggest a link between long-term exposure to ionizing radiation and increased risks of mortality from CVD in the people living in areas contaminated by radiation from the Semipalatinsk nuclear test site, depending on the radiation dose. The highest relative risks of mortality were observed for cerebrovascular diseases in general, hemorrhagic strokes, peripheral arterial atherosclerosis, congenital heart defects, and ischemic strokes. Calculation of the risk ratio for CVD revealed a direct dose–response relationship: in the dose range of 100 to 500 mSv, mortality risks increased by 3.14 times, and at doses greater than 500 mSv, they increased by 7.05 times. Analysis of dynamic series of intensive mortality rates from CVD and their forecasting showed that mortality rates remain stable in both the exposed and unexposed groups until 2050. This indicates that not only people directly exposed to ionizing radiation but also their descendants in subsequent generations will be at increased risk of mortality from CVD now and in the future. The primary healthcare objective for this category of Kazakhstani citizens is the active implementation of preventive measures aimed at reducing these risks.

## Figures and Tables

**Table 1:** Structure of overall mortality in the exposed and unexposed study groups.

Cause of Death According to ICD 10	Exposed Group (N = 8580)	Control Group (N = 3743)	All Patients (N = 12,323)	Test Statistic *
Infections	475 (5.5%)	144 (3.8%)	619 (5.0%)	Χ^2^ = 189.3 *p* < 0.01
Neoplasms	1687 (19.7%)	499 (13.3%)	2186 (17.7%)	
Hematological diseases	18 (0.2%)	20 (0.5%)	38 (0.3%)	
Diseases of the endocrine system	64 (0.7%)	36 (1.0%)	100 (0.8%)	
Mental illnesses	9 (0.1%)	1 (0.0%)	10 (0.1%)	
Diseases of the nervous system	156 (1.8%)	28 (0.7%)	184 (1.5%)	
CVD	3482 (40.6%)	1886 (50.4%)	5368 (43.6%)	
Respiratory diseases	1140 (13.3%)	374 (10.0%)	1514 (12.3%)	
Gastrointestinal diseases	286 (3.3%)	102 (2.7%)	388 (3.1%)	
Skin diseases	7 (0.1%)	3 (0.1%)	10 (0.1%)	
Diseases of the musculoskeletal system	5 (0.1%)	1 (0.0%)	6 (0.0%)	
Diseases of the genitourinary system	101 (1.2%)	43 (1.1%)	144 (1.2%)	
Pregnancy-related cases	33 (0.4%)	6 (0.2%)	39 (0.3%)	
Perinatal diseases	120 (1.4%)	70 (1.9%)	190 (1.5%)	
Congenital malformations, excluding congenital heart disease	42 (0.5%)	9 (0.2%)	51 (0.4%)	
Other causes and deviations	156 (1.8%)	68 (1.8%)	224 (1.8%)	
Injuries	520 (6.1%)	317 (8.5%)	837 (6.8%)	
External causes	67 (0.8%)	36 (1.0%)	103 (0.8%)	
Suicide	212 (2.5%)	100 (2.7%)	312 (2.5%)	

* Pearson’s Chi-squared test.

**Table 2:** Dynamics of the structure of mortality from CVD by age groups and study periods.

Characteristics	Exposed Group, n = 3482	Control Group, n = 1886	*p*
Age groups			
0–9	43 (1.2%)	44 (2.3%)	Χ^2^ = 26.32, *p* < 0.01
10–19	13 (0.4%)	12 (0.6%)	
20–29	32 (0.9%)	15 (0.8%)	
30–39	77 (2.2%)	51 (2.7%)	
40–49	221 (6.3%)	134 (7.1%)	
50–59	369 (10.6%)	234 (12.4%)	
60–69	853 (24.5%)	405 (21.5%)	
70–79	942 (27.1%)	538 (28.5%)	
>80	932 (26.8%)	453 (24.0%)	
Research periods			
1949–1954	42 (1.2%)	41 (2.2%)	Χ^2^ = 219.77, *p* < 0.01
1955–1959	45 (1.3%)	29 (1.5%)	
1960–1964	218 (6.3%)	21 (1.1%)	
1965–1969	239 (6.9%)	62 (3.3%)	
1970–1974	274 (7.9%)	97 (5.1%)	
1975–1979	338 (9.7%)	139 (7.4%)	
1980–1984	343 (9.9%)	235 (12.5%)	
1985–1989	278 (8.0%)	177 (9.4%)	
1990–1994	290 (8.3%)	214 (11.3%)	
1995–1999	245 (7.0%)	264 (14.0%)	
2000–2004	303 (8.7%)	156 (8.3%)	
2005–2009	325 (9.3%)	180 (9.5%)	
2010–2014	172 (4.9%)	77 (4.1%)	
2015–2019	145 (4.2%)	91 (4.8%)	
2020–2024	225 (6.5%)	103 (5.5)	

**Table 3:** Dynamics of intensive mortality rates from CVD (per 100,000 population) by study periods.

Periods	Exposed Group 95% CI	Control Group 95% CI	RR 95% CI
1949–1954	152.96	168.06	0.9
	106.70–199.22	116.61–219.50	0.59–1.39
1955–1959	145.65	107.015	1.36
	103.09–188.21	68.06–145.96	0.85–2.17
1960–1964	642.71	73.86	8.7
	557.38–728.02	42.27–105.45	5.5–13.6
1965–1969	641.78	201.97	3.2
	560.41–723.14	151.69–252.24	2.4–4.2
1970–1974	699.23	303.14	2.3
	616.28–782.17	242.81–363.47	1.8–2.9
1975–1979	843.003	427.72	1.97
	753.16–932.92	356.61–498.82	1.62–2.4
1980–1984	773.74	655.51	1.18
	691.86–855.63	571.69–739.32	1.0–1.4
1985–1989	648.2	466.40	1.38
	572.00–724.39	397.69–535.11	1.15–1.67
1990–1994	647.47	519.22	1.24
	572.95–721.99	449.71–588.87	1.04–1.48
1995–1999	532.31	577.1	0.92
	465.66–598.98	507.48–646.71	0.77–1.09
2000–2004	741.56	485.68	1.5
	658.06–825.06	409.46–561.89	1.26–1.85
2005–2009	868.24	981.30	0.88
	773.84–962.63	837.94–1124.65	0.73–1.06
2010–2014	469.25	445.26	1.05
	399.12–539.38	345.81–544.72	0.8–1.37
2015–2019	420.64	592.1	0.7
	352.18–489.11	470.44–713.76	0.54–0.92
2020–2024	702.95	748.06	0.93
	611.1–794.8	603.59–892.53	0.74–1.87

**Table 4:** CVD mortality structure in the study groups.

Cause of Death (ICD-10)	Exposed Group (n, %)			Control Group (n, %)			
	1949–1969, n = 544	1970–1994, n = 1527	>1995, n = 1437	1949–1969, n = 153	1970–1994, n = 864	>1995, n = 1078	p *
Chronic ischemic heart disease (I25)	237 (43.6%)	665 (43.7%)	592 (41.8%)	81 (52.9%)	477 (55.3%)	464.0 (53.3%)	P1 = 0.04 P2 < 0.0001 P3 < 0.0001
Acute myocardial infarction (I21)	113 (20.8%)	265 (17.4%)	206 (14.6%)	22 (14.4%)	163 (18.9%)	161.0 (18.5%)	P1 = 0.09 P2 = 0.34 P3 = 0.92
Hypertension (I10–I15)	13 (2.4%)	31 (2.0%)	156 (11.0%)	7 (4.6%)	43 (5.0%)	74.0 (8.5%)	P1 = 0.24 P2 < 0.0001 P3= 0.7
Chronic cerebrovascular diseases (I60–I69)	2 (0.4%)	97 (6.4%)	108 (7.6%)	1 (0.7%)	10 (1.2%)	9.0 (1.0%)	P1 = 0.04 ** P2 < 0.0001 P3 = 0.02
Ischemic stroke (I60–I61)	61 (11.2%)	152 (10.0%)	91 (6.4%)	14 (9.2%)	68 (7.9%)	75 (8.6%)	P1 = 0.52 P2 = 0.09 P3 = 0.81
Hemorrhagic stroke (I63)	74 (13.6%)	172 (11.3%)	170 (12.0%)	14 (9.2%)	40 (4%)	63 (7.2%)	P1 = 0.2 P2 < 0.0001 P3 = 0.01
Peripheral atherosclerosis (I70)	1 (0.2%)	61 (4.0%)	13 (0.9%)	2 (1.3%)	4 (0.5%)	7 (0.8%)	P1 = 0.12 ** P2 < 0.0001 ** P3 = 0.68
Congenital heart defects (Q20–Q28)	8 (1.5%)	21 (1.4%)	15 (1.1%)	2 (1.3%)	10 (1.2%)	2 (0.2%)	P1 = 1.0 ** P2 = 0.8 ** P3 = 0.006 **
Other heart diseases I95–I99	35 (6.4%)	59 (3.9%)	64 (4.5%)	10 (6.5%)	47 (5.5%)	16 (1.8%)	P1= 1.0 P2 = 0.09 P3 = 0.1

P1 = 1949–1969, P2 = 1970–1994, P3 > 1995, * χ^2^ with Yates’ amendment, ** two-tailed Fisher test.

**Table 5:** Risk ratios of CVD mortality depending on the equivalent dose of radiation and time from irradiation.

Characteristics	Variable	n (%)	HR (Multivariable) (95% CI)
Dose group	<100 mSv (reference value)	4923 (39.9)	-
	100–500 mSv	1152 (9.3)	3.14 (2.7–3.66) (*p* < 0.001)
	>500 mSv	6248 (50.7)	7.05 (5.24–9.49) (*p* < 0.001)
Mortality by periods	1949–1969 (reference value)	2604 (21.8)	-
	1970–1994	5162 (43.3)	1.46 (1.33–1.63) (*p* < 0.001)
	After 1995	4154 (34.8)	1.5 (1.36–1.65) (*p* < 0.001)

## Data Availability

The raw data supporting the conclusions of this article will be made available by the authors on request.
